# A Practical Data-Gathering Algorithm for Lossy Wireless Sensor Networks Employing Distributed Data Storage and Compressive Sensing

**DOI:** 10.3390/s18103221

**Published:** 2018-09-24

**Authors:** Ce Zhang, Ou Li, Guangyi Liu, Mingxuan Li

**Affiliations:** National Digital Switching System Engineering and Technological R&D Center, Zhengzhou 450002, China; cezhang@foxmail.com (C.Z.); zzliou@126.com (O.L.); seu_lmx@foxmail.com (M.L.)

**Keywords:** WSNs, CS, distributed data storage, packet loss rate, energy efficiency

## Abstract

Reliability and energy efficiency are two key considerations when designing a compressive sensing (CS)-based data-gathering scheme. Most researchers assume there is no packets loss, thus, they focus only on reducing the energy consumption in wireless sensor networks (WSNs) while setting reliability concerns aside. To balance the performance–energy trade-off in lossy WSNs, a distributed data storage (DDS) and gathering scheme based on CS (CS-DDSG) is introduced, which combines CS and DDS. CS-DDSG utilizes broadcast properties to resist the impact of packet loss rates. Neighboring nodes receive packets with process constraints imposed to decrease the volume of both transmissions and receptions. The mobile sink randomly queries nodes and constructs a measurement matrix based on received data with the purpose of avoiding measuring the lossy nodes. Additionally, we demonstrate how this measurement matrix satisfies the restricted isometry property. To analyze the efficiency of the proposed scheme, an expression that reflects the total number of transmissions and receptions is formulated via random geometric graph theory. Simulation results indicate that our scheme achieves high precision for unreliable links and reduces the number of transmissions, receptions and fusions. Thus, our proposed CS-DDSG approach effectively balances energy consumption and reconstruction accuracy.

## 1. Introduction

As the perceptual layer of the Internet of Things (IoT) [1,2], wireless sensor networks (WSNs) [3] are widely deployed for purposes such as environment monitoring [4], industry automation [5] and military reconnaissance [6]. WSNs consist of many sensors and play a key role in sensing and gathering data from the surrounding environment. Because of harsh environments and energy-limited nodes, there are two key considerations in WSNs design: reliability and energy efficiency. In addition, nodes that are closer to the sink require more forwarding tasks than others, resulting in higher energy consumption as well as a reduction in the lifetime of the entire network.

Compressive sensing (CS) theory [7,8] provides a new method for reducing communication energy consumption. CS points out that, for the compressible signals in WSNs, a small collection of linear projections is sufficient to achieve near-perfect reconstruction, which reduces energy consumption and prolongs network lifetime. Thus, a considerable amount of research has been conducted concerning ways to utilize CS to gather data in WSNs. The CS-based data-gathering schemes in [9,10,11] obtained the member node readings utilizing fixed routing, in which ordinary nodes forward compressed data to the static sink node through multi-hops. Lou et al. [9] and Lou et al. [10] combined CS and routing protocols to reduce the number of transmissions. In [11,12,13,14,15], the use of sparse measurement matrix is investigated to reduce the number of nodes involved in data gathering. Introducing CS effectively reduces the energy required for communication and distributes energy consumption loads more evenly. However, if a parent node (which holds a combination of child node readings) loses its packet, then all the information from the child nodes is also lost. Hence, unreliable links have a serious impact on data gathering and make it difficult to reliable gather data reliably through a centralized sink node. Additionally, Kong et al. [16] reported that unreliable links are widespread in WSNs, where the average packet loss rate is 40–50%. Thus, assuming completely reliable links is unfeasible and oversimplifies the problem.

To resolve this problem, distributed data storage (DDS) [17,18,19] is proposed to enable reliable data gathering by employing redundancy. In contrast to a centralized sink, a mobile sink collects data from a small subset of the total nodes to recover all the data. It is worth mentioning that DDS effectively reduces the impact of packet loss on data gathering because there is no static routing, although few researchers have focused on this advantage. However, DDS requires a large number of transmission tasks to ensure sufficient redundancy, which is potentially catastrophic for nodes with energy limitations. Thus, it is imperative to investigate effective ways to apply DDS for data gathering with the dual purposes of resisting packet loss and reducing the number of transmissions.

To address this problem, many studies have been carried out on this topic. In [20,21,22], CS is combined with DDS to exploit the advantages of both technologies. The goal of Talari et al. [20] was to reduce the number of transmissions by exploiting the spatial correlations of nodes based on CS with the broadcast properties of wireless channels. In this scheme, the nodes store received data and broadcast the data with a given probability. The performance of data reconstruction was further improved in [21]. Yang et al. [21] found that the number of receptions was higher than the number of transmissions. Hence, Yang et al. [21] focused on reducing the total number of both transmissions and receptions simultaneously. In [22], both the spatial and temporal correlations of nodes are exploited to reduce the number of transmissions. All the above studies take advantage of broadcast routing and consider how to reduce the transmission energy cost. However, compared with fixed routing, such as tree routing and cluster routing, broadcasting data consumes more reception energy because neighboring nodes receive broadcast data whether they need it or not. For example, in [20,21,22], the neighboring nodes first receive the broadcasting data and then determine whether to merge the data based on certain conditions. Consequently, broadcasting data consumes large amount of reception energy, although the received data are rarely merged. Furthermore, none of these studies consider the problem of packet loss; instead, they make the unrealistic assumption that the wireless links are completely reliable.

Tackling the abovementioned consideration, two challenges must be resolved. The first involves how to effectively reduce the quantity of data disseminated (transmissions and receptions), especially the number of receptions rather than the number of fusions. The second problem is related to reducing the impact of lossy links (namely, the packet loss rate) on data reconstruction. To solve these two challenges, a distributed data storage and gathering algorithm based on compressive sensing (CS-DDSG) is proposed utilizing CS and DDS. Relying on collected data, the mobile sink generates a sparse measurement matrix aimed at reducing communication energy consumption. Furthermore, it is proven that the measurement matrix satisfies the restricted isometry property (RIP) [23]. Based on random geometric graph theory, an expression of the total number of transmissions and receptions is formulated to analyze the energy consumption of CS-DDSG.

The reminder of this paper is organized as follows. In Section 2, we commence by reviewing the CS theory and introduce the network model. In Section 3, we present the proposed CS-DDSG algorithm, describe the formulation of the measurement matrix and provide a proof that this matrix can satisfy RIP. Based on the proposed scheme, we formulate the expression of the total number of transmissions and receptions in Section 4. We present our simulations and their results and investigate the performance of CS-DDSC in Section 5. Finally, concluding remarks are provided in Section 6.

## 2. Preliminaries and Network Model

In this section, we introduce CS theory and then describe the network model and our motivation.

### 2.1. Compressed Sensing

In WSNs, assume that N sensor readings are denoted by $X={\left(x_{1},\cdots ,x_{N}\right)}^{T}$, where $x_{i},i\in \left[1,N\right]$ denotes the reading of node i with K-sparse representation at a basis $\Psi \in {\mathbb{R}}^{N\times N}$:

(1) X=Ψθ, 

where $\theta \in {\mathbb{R}}^{N}$ is a coefficient vector corresponding to the sparse basis Ψ. X is K-sparse and compressive if the vector θ has at most K(K≤N) nonzero coefficients or (N−K) smallest coefficients can be ignored.

We assume the measurement matrix is $\Phi \in {\mathbb{R}}^{M\times N}$ and is uncorrelated with the basis Ψ, then the CS measurements of X can be expressed as follows:

(2) Y=ΦX=ΦΨθ=Θθ, 

where M≪N and Θ=ΦΨ is a sensing matrix. The original signal X can be reconstructed with an overwhelming probability from M measurements by $l_{1}$-norm minimization as follows:

(3) $$\begin{array}{l} \min:\hat{X}=\min{\left\|X\right\|}_{1}\;\\ \;s.t.\;:Y=\Phi X, \end{array}$$

where $\hat{X}$ denotes the reconstructed sparse signal of X.

To reconstruct X, two factors must be considered: (1) X is compressive at Ψ; and (2) Φ must satisfy the RIP with M≥cklg(N/k). Therefore, K-sparse X satisfies the following condition:

(4) $$\left(1-\varepsilon \right){\left\|\theta \right\|}_{2}^{2}\leq {\left\|\Phi \theta \right\|}_{2}^{2}\leq \left(1+\varepsilon \right){\left\|\theta \right\|}_{2}^{2},\;$$

where c,ε∈(0,1), while Φ satisfies RIP with the parameter ε.

### 2.2. Network Model

We consider a single-sink WSN consisting of N battery-powered sensors. The sensors are deployed in a square area with a boundary length of 1. We assume all nodes have an identical transmission radius of $r_{t}$, and that any two nodes can communicate with each other if their Euclidian distance d satisfies $d\leq r_{t}$. To guarantee the network connectivity, $r_{t}$ should also satisfy the following condition [24]:

(5) $$r_{t}^{2}>S\cdot \mathrm{In}\left(N\right)/\left(\pi N\right),\;$$

where S denotes the deployment area and S=1×1. Let $X_{N\times 1}={\left(x_{1},\cdots ,x_{N}\right)}^{T}$ denotes the N node readings. Since the readings are spatiotemporally correlative with each other, X can be compressed on an orthogonal basis $\Psi ={\left(\phi_{i,j}\right)}_{N\times N}$. The fast Fourier transform (FFT) orthonormal basis is adopted as the sparse representation basis in this paper. Let $\Phi ={\left(\varphi_{i,j}\right)}_{M\times N}$ denote the measurement matrix. The measurement vector $Y\in {\mathbb{R}}^{M\times 1}$ can be computed with Equation (2). Furthermore, we introduce the expression of Φ in Section 3. Thus, the CS-DDSG network model coincides with the CS model.

In addition, we define the normalized mean absolute error (NMAE) metric to evaluate the accuracy of reconstruction accuracy:

(6) $$\mathrm{NMAE}=\frac{{\left\|\hat{X}-X\right\|}_{2}}{{\left\|X\right\|}_{2}}=\frac{\sqrt{\sum_{n=1}^{N}{\left({\hat{x}}_{n}-x_{n}\right)}^{2}}}{\sqrt{\sum_{n=1}^{N}x_{n}{}^{2}}},\;$$

Equation (6) shows that the smaller the NMAE is, the better performance the algorithm can achieve.

### 2.3. Motivation

**Figure 1:**
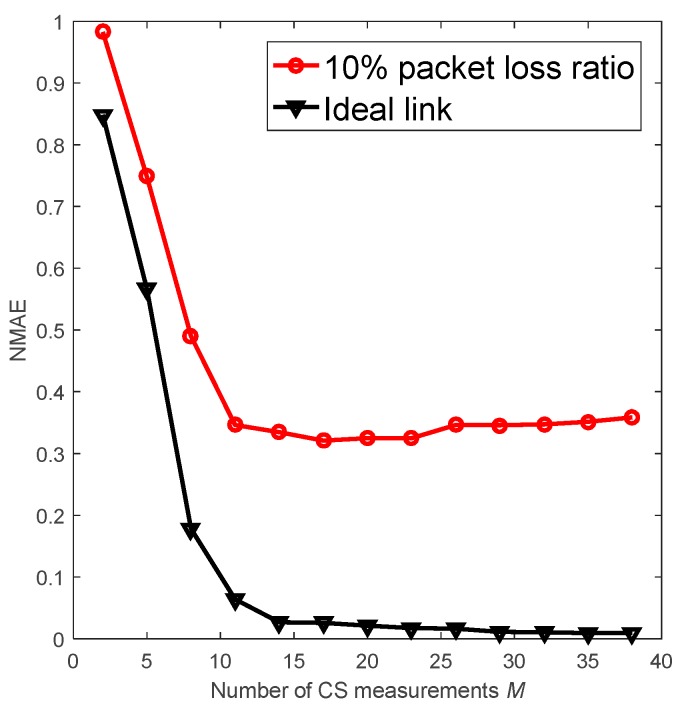
Performance of CDG with ideal link and lossy link.

**Figure 2:**
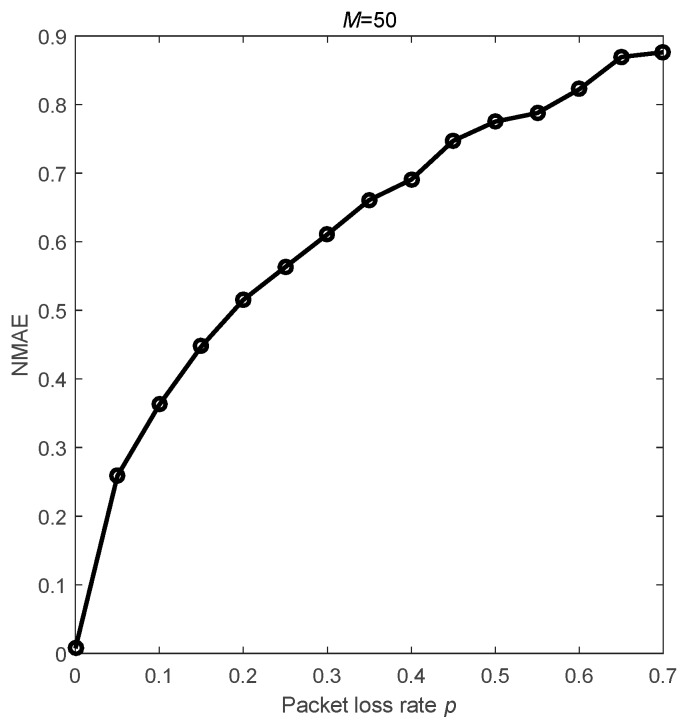
The relationship between the packet loss rate and the NMAE.

In this subsection, we investigate the impact of packet loss on the CS recovery performance relying on the fixed routing. Figure 1 presents the performance of the CDG [9] algorithm with cluster topology in unreliable links. In this scheme, there are 100 nodes and the member nodes forward the packets to the cluster head via a one-hop route. When the packet loss rate is 10%, the recovery accuracy is worse than the accuracy in the ideal link. Furthermore, increasing the measurements cannot improve the algorithm’s performance. For M=50 measurements, Figure 2 indicates that the accuracy declines with the increase of packet loss rate.

We consider one of the clusters containing $N_{1}$ nodes. For the CDG algorithm with fixed routing, the cluster head receives the data vector $X_{N_{1}\times 1}={\left(x_{1},\cdots ,x_{i},\cdots ,x_{N_{1}}\right)}^{T}$ in reliable links. The measurements Y can be represented as

(7) $$Y=\left(\begin{array}{c} y_{1}\\ y_{2}\\ \vdots \\ y_{M} \end{array}\right)=\left(\begin{array}{ccc} \phi_{11} & \cdots & \phi_{1N_{1}}\\ \vdots & & \vdots \\ \phi_{M1} & \cdots & \phi_{MN_{1}} \end{array}\right)\left(\begin{array}{c} x_{1}\\ \begin{array}{c} \vdots \\ x_{i}\\ \vdots \end{array}\\ x_{N_{1}} \end{array}\right).\;$$

If the packet of node i is missing due to unreliable links, then its cluster head will receive $X_{N_{1}\times 1}^{\prime }={\left(x_{1},\cdots ,{x^{\prime }}_{i},\cdots ,x_{N_{1}}\right)}^{T}$ and the measurement Y can be represented as

(8) $$Y^{\prime }=\left(\begin{array}{c} {y^{\prime }}_{1}\\ {y^{\prime }}_{2}\\ \vdots \\ {y^{\prime }}_{M} \end{array}\right)=\left(\begin{array}{ccc} \phi_{11} & \cdots & \phi_{1N_{1}}\\ \vdots & & \vdots \\ \phi_{M1} & \cdots & \phi_{MN_{1}} \end{array}\right)\left(\begin{array}{c} x_{1}\\ \begin{array}{c} \vdots \\ {x^{\prime }}_{i}\\ \vdots \end{array}\\ x_{N_{1}} \end{array}\right).\;$$

According to Equations (7) and (8), one missing packet affects every element $y_{i}$ of the measurement vector. Thus, the sink recovers all the data X using $Y^{\prime }$ and Φ, which leads to an imprecise or invalid reconstruction. Furthermore, the accuracy is even worse under tree-based routing. This deficiency occurs because if one packet of a parent node is missing, then all the information from its child nodes is lost too. Additionally, simply increasing the number of measurements or the number of retransmissions does not help much in improving the recovery accuracy. Therefore, the CS-based algorithm is sensitive to packet loss. In the next section, we investigate how to resist unreliable links, while using fewer transmissions and receptions by utilizing broadcasting properties.

## 3. Proposed CS-DDSG Scheme

### 3.1. Procedures of CS-DDSG

**Figure 3:**
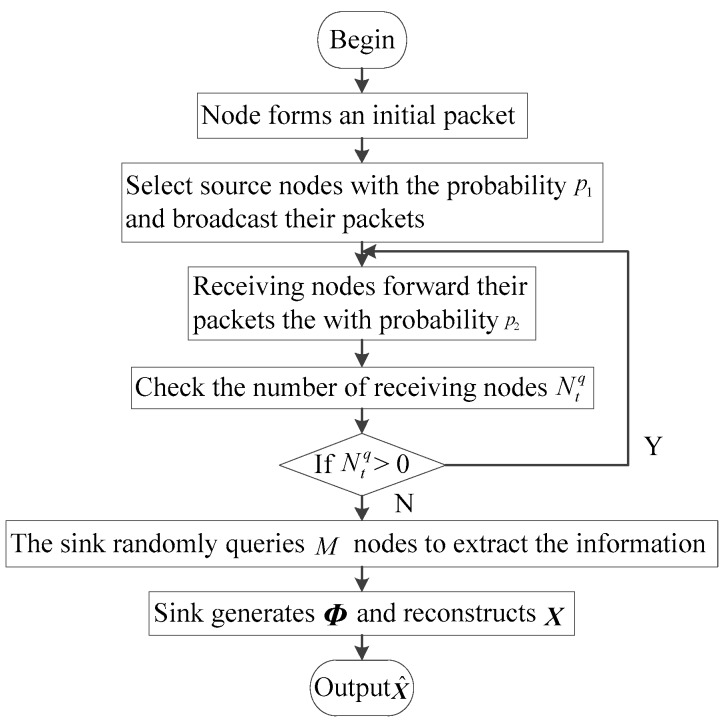
Flow chart of CS-DDSG.

Based on the network model, we propose CS-DDSG to avoid packet loss and reduce the total number of transmissions and receptions, as presented in Figure 3. The procedures involved in CS-DDSG are detailed below.

Stage 1. Initialization. The proposed scheme requires precise time to help nodes to cooperate with each other. Assuming the network is synchronized and slotted based on Reference Broadcast Synchronization (RBS) [25], which can achieve the goal of high accuracy and energy-efficiency. At the beginning of data gathering, each node senses a data $x_{i}$ and generates a coefficient $\varphi_{i}=1$. Then, each node i forms an initial packet, denoted by S(i) which defines has two components:

(9) $$S\left(i\right)=\left\{ \begin{array}{l} S\left(i\right).\mathrm{id}=[i]\\ S\left(i\right).\mathrm{data}=x_{i} \end{array}\right..\;$$

The component S(i).id stores the node ID of nodes and S(i).data stores the readings.

Stage 2. Broadcasting. After a fixed and long enough period of time for synchronization and initialization, $N_{s},\left(N_{s}<N\right)$ nodes are randomly selected as source nodes with a probability $p_{1}$ in this stage. The source nodes broadcast their own packets and do not receive any packets. If an ordinary node m(m∈[1,N]) is located with the communication range of the source node n(n∈[1,N]) and has not received a packet before, then node m receives the data broadcasted by node n and updates its packet as follows:

(10) $$S\left(m\right)=\left\{ \begin{array}{l} S\left(m\right).\mathrm{id}=[m,n]\\ S\left(m\right).\mathrm{data}=x_{m}+x_{n} \end{array}\right..\;$$

If node m has already received any other broadcast data, then this node stops receiving data; in other words, each node receives only one broadcast packet.

Stage 3. Forwarding. In the following, only the receiving nodes from Stage 2 continue to broadcast their updated packets to neighboring nodes with the probability $p_{2}$. Similarly, the neighboring nodes around the forwarding nodes will receive a packet only if they have not received any prior packets. These new receiving nodes broadcast their updated packets as described above. Actually, the Stage 2 and Stage 3 could start simultaneously. Nodes get the packets of source nodes in Stage 2 and then decide whether to broadcast immediately. Thus, the neighboring nodes of those forwarding nodes could update their packets relying on the packets of source nodes or forwarding nodes. Finally, the forwarding operation will stop until there are no new reception nodes. Because of the reception condition and the small probability $p_{2}$, in practice, the forwarding process stops after repeating only a few times, which is analyzed in Section 5 in detail.

Stage 4. Visiting. The mobile sink starts the visiting phase after a fixed and sufficiently long period, which can be preset according to the number of nodes N. M nodes are randomly queried by the mobile sink to extract the corresponding information, i.e., the measurement vector Y and the measurement matrix Φ. Finally, the entire network’s readings X can be reconstructed from Y and Φ based on Equation (3). The entire pseudocode of CS-DDSG is presented in Algorithms 1 and 2.

**Algorithm 1** The CS-DDSG algorithm
**Input:**
 The probability of selecting source nodes: *P*_1_; The probability of forwarding: *P*_2_; The number of measurements: *M*;
**Output:**
 Measurement vector: ***y***; Measurement matrix: ***Φ***;
**Stage 1:**
1: **for**
*i* = 1:*N*2: *S*(*i*).id = [*i*];3: *S*(*i*).*data* = *x_i_*;4: **end for**
**Stage 2:**
5: Nodes select themselves with the probability *p*_1_ and broadcast their packets;6: *N*_2_ = 0;7: **for**
*i* = 1:*N*·*p*_1_8: **for**
*j* = 1:*N*9: **if** node *i* receives the broadcasting data from node *j*10: *S*(*j*).*id* = [*j,i*];11: *S*(*j*).*data* = *x_j_* + *x_i_*;12: *N*_2_ = *N*_2_ + 1;13: **end if**14: **end for**15: **end for**
**Stage 3:**
16: The receiving nodes in Stage 2 forward their update packets with probability *p*_2_.17: *N*_3_ = *N*_2_*p*_2_;18: **for**
*loop* = 1:max19: if *N*_3_ ≤ 120: break21: **end if**22: **if** node *j* forwards its packets23: for *i* = 1: *N*24: **if** node *i* has not received a packet and hears node *j*25: *S*(*i*).*id* = [*i,j*];26: *S*(*i*).*data* = *x_i_* + *x_j_*;27: *N*_3_ = *N*_3_ + 1;28: **end if**29: **end for**30: **end if**31: The reception nodes in the stage 3 forwarding their packets with probability *p*_2_.32: **end for**
**Stage 4:**
33: The mobile sink queries *M* nodes to generate ***Φ*** and ***Y*** .34: ***Φ*** = ***zeros*** (*M*, *N*)35: **if** node *i_k_* are queried36: ***Ω**_k_* = *S*(*i_k_*).id;37: ***Φ***(*k*, ***Ω**_k_*) = 1;38: **end if**39: Return ***Φ*** and ***Y***.

**Algorithm 2** CS Reconstruction
**Input:**
 Measurement vector: ***y***; Measurement matrix: ***Φ***;
**Output:**
 Reconstructed vector: $\hat{X}$1: Sink creates ***Y*** and BDM ***Φ*** based on ***y**_i_* and ***Φ***_*i*_;2: $\hat{\theta }$ = arg min ||***θ***||_1_ s.t. ***Y*** = ***ΦΨθ***;3: $\hat{X}=\Psi \hat{\theta }$

### 3.2. Selection of Parameters

In this subsection, we investigate the values of the parameters $r_{t}$ and $p_{2}$. We consider a network with N=400 nodes, which are randomly deployed over an area of size S=1×1 in this paper. As described in Section 2, to ensure the network connectivity, $r_{t}$ must satisfy the condition in Equation (5). Thus, $r_{t}>0.069$; we set $r_{t}=0.075$.

**Figure 4:**
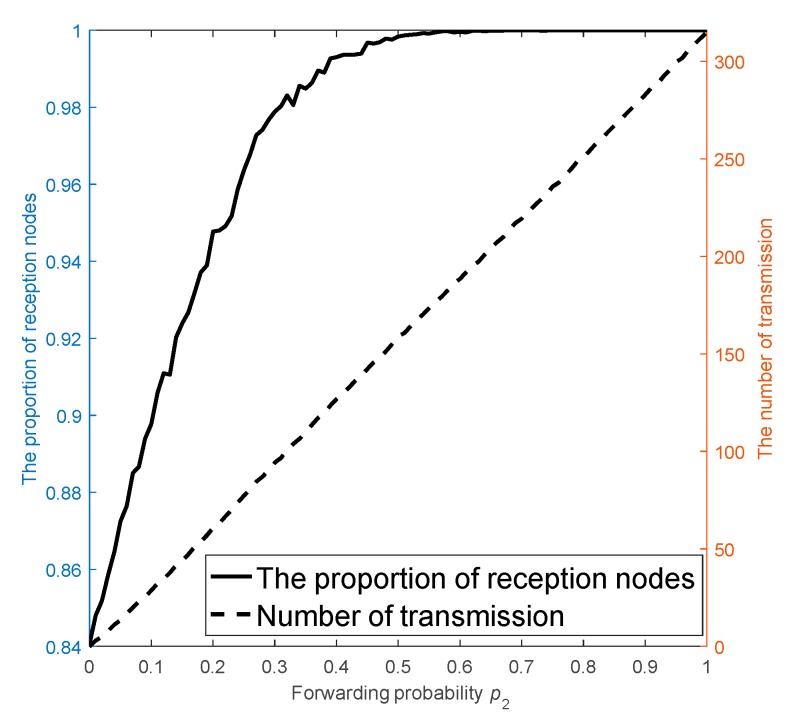
The impact of forwarding probability on the number of transmitting and receiving nodes.

In Stage 3 of CS-DDSG, nodes forward their updated packets with a probability $p_{2}$ and all neighboring nodes can receive this data. For the sake of an appropriate $p_{2}$ that reduces the number of transmissions $N_{t}$ and increases the proportion of reception nodes $P_{r}$ simultaneously, we simulate $N_{r}$ and $P_{r}$ versus $p_{2}$ by setting $p_{1}=0.2$ and $r_{t}=0.075$ as shown Figure 4, where all normal nodes stop receiving any data after merging one packet. As Figure 4 shows, as $p_{2}$ increases, the values of $N_{t}$ and $P_{r}$ both increase. Furthermore, $P_{r}$ increases almost linearly with $p_{2}$. Thus, when $p_{2}=0.32$, 98% nodes receive a broadcast packet. Moreover, as $p_{2}$ increase beyond 0.32, $N_{r}$ increases less, while $P_{r}$ increases sharply. Therefore, the appropriate value for $p_{2}$ is 0.32, because that value provides a balanced trade-off between the number of transmissions and the percentage of receiving nodes.

### 3.3. Measurement Matrix Formulation

In this subsection, we present the formulation procedure for the measurement matrix. As we introduced above, in Stage 4, after the mobile sink queries the M nodes, which are denoted by $\left(n_{i_{1}},n_{i_{2}}\cdots ,n_{i_{k}},\cdots n_{i_{M}}\right),i_{1}<i_{2}<\cdots <i_{M},i_{k}\in \left[1,N\right]$, the measurement matrix Φ is constructed based on the M packets. Suppose $\Omega_{k}$ is the index of node ID and its definition is expressed as follows:

(11) $$\Omega_{k}=S\left(n_{i_{k}}\right).\mathrm{id}.\;$$

Initially, Φ is an all-zero M×N matrix, then Φ is formulated at this step which is given by Equation (12):

(12) $$\Phi \left(k,j\right)=\left\{ \begin{array}{l} 1,\;j=\Omega_{k}\\ 0,\;otherwise \end{array}\right..\;$$

For example, assume there are five nodes in the network (i.e., N=5). If the mobile sink queries two nodes (i.e., M=2), then Φ can initially be expressed as follows:

(13) $$\Phi_{2\times 5}=\left(\begin{array}{ccccc} 0 & 0 & 0 & 0 & 0\\ 0 & 0 & 0 & 0 & 0 \end{array}\right).\;$$

Suppose that nodes 2 and 4 are selected by the sink, and their packets components are as follows:

(14) $$\begin{array}{l} S\left(2\right).\mathrm{id}=\left[2,5\right]\\ S\left(4\right).\mathrm{id}=\left[1,4\right], \end{array}$$

then $\varphi_{1,2}=\varphi_{1,5}=1$ and $\varphi_{2,1}=\varphi_{2,4}=1$. Finally, the matrix Φ becomes:

(15) $$\Phi_{2\times 5}=\left(\begin{array}{ccccc} 0 & 1 & 0 & 0 & 1\\ 1 & 0 & 0 & 1 & 0 \end{array}\right).\;$$

Moreover, the measurement vector Y is expressed as follows:

(16) $$Y={\left(S\left(2\right).data,S\left(4\right).data\right)}^{T}.\;$$

Obviously, Φ is a sparse matrix, whose sparsity degree is influenced by p, $p_{1}$ and $p_{2}$. Furthermore, Equation (12) indicates that Φ is constructed by relying on the gathered data, which precludes the need to measure lost data. Thus, Y is not influenced by lost packets at all. Therefore, CS-DDSG is resistant to the packet loss rate.

### 3.4. Does the Measurement Matrices Satisfy RIP?

The structure of measurement matrix Φ is random and relies on the receiving nodes. Thus, CS-DDSG avoids measuring the lost nodes and avoids the packet loss. The question is: Does Φ obey RIP to utilize the CS theory? Unfortunately, it is an NP-hard problem to prove the RIP property of a matrix. However, Yang et al. [21] reported that recovery performance can be guaranteed with high probability when the rows of the measurement matrix are linearly independent. We investigate this proposition below.

The rows of Φ are linearly dependent when one of the following two situations occurs.

**Case** **1.** *Any row $\varphi_{k}$ can be expressed as a linear combination of other rows*.

**Proof.**  The measurement coefficient is 1; thus, if $\varphi_{k}$ can be expressed as a linear combination of rows $\varphi_{k_{1}},\cdots ,\varphi_{k_{q}}$, q=2,⋯,N−1, they satisfy the following:

(17) $$\varphi_{k}=\varphi_{k_{1}}+\cdots +\varphi_{k_{q}}.\;$$

Suppose $I_{k}=\left\{j|\varphi_{k,j}\neq 0\right\}$ and $I_{k_{i}}=\left\{j|\varphi_{k_{i},j}\neq 0\right\}$; if the condition of Equation (17) is satisfied, then $I_{k}=\cup_{i=1}^{q}I_{k_{i}}$ and we can obtain

(18) $$\left|I_{k}\right|>\left|I_{k_{i}}\right|,i\in \left[1,q\right],\;$$

where |·| denotes the number of elements in the set. Thus, Equation (17) can be satisfied when one of the following two situations occurs. The first situation would occur if node k were to receive packets from nodes $k_{1},\cdots ,k_{q}$ and merge their packets. However, this situation contradicts the reception condition under which each node receives one packet. Thus, the condition of Equation (17) cannot occur.

The second situation would occurs when node $k_{2}$ receives a packet from node $k_{1}$ and node $k_{3}$ receives a packet from node $k_{2}$. It follows that node $k_{q}$ receives a packet from node $k_{q-1}$. Finally, node k receives the packet from node $k_{q}$. According to the condition in Equation (10), $I_{k_{q}}$ satisfies the following:

(19) $$I_{k_{q}}=\left\{k_{q}\right\}\cup \left(\cup_{i=1}^{q-1}I_{k_{i}}\right).\;$$

After node k updates its packet, $I_{k}$ satisfies:

(20) $$I_{k}=\left\{k\right\}\cup I_{k_{q}}.\;$$

Obviously, $k\in I_{k}$ but $k\notin I_{k_{q}}$. Thus, $I_{k}=\left\{k\right\}\cup \left(\cup_{i=1}^{q}I_{k_{i}}\right)$ and Equation (17) is false.

Consequently, it can be concluded that no rows can be linearly expressed by other rows.  □

**Case** **2.** *Any two rows $\varphi_{i}$ and $\varphi_{j}$ are linearly dependent*.

**Proof.**  $\varphi_{i}$ and $\varphi_{j}$ are linearly dependent if and only if they are precisely the same. However, according to the reception condition, each node receives only one packet and merges with its own unique packet. Therefore, although node i and node j may receive the same broadcasting packet from a common neighboring node, their packets will still be different. Therefore, none of the rows are linearly dependent.  □

In conclusion, the rows of the measurement matrix Φ are linearly independent; consequently, in CS-SSDG, X can be reconstructed from Y with a very high probability.

## 4. Formulating the Expression of the Total Number of Transmissions and Receptions

Compared with the mainstream algorithms [15,20,21], the proposed scheme CS-DDSG reduces the number of transmissions and receptions rather than the number of fusions. In this section, we formulate the total number of transmissions $N_{Ttot}$ and receptions $N_{Rtot}$ based on the random geometric graph (RGG) mode [26] and the torus convention [27] to investigate the efficiency in reducing $N_{Ttot}$ and $N_{Rtot}$.

According to Section 3, $N_{Ttot}$ and $N_{Rtot}$ can be expressed as follows:

(21) $$\begin{array}{l} N_{Ttot}=N_{t}^{П}+N_{t}^{Ш}=N_{s}+\sum_{q=1}^{N_{f}}N_{t}^{q}\\ N_{Rtot}=N_{r}^{П}+N_{r}^{Ш}=N_{r}^{П}+\sum_{q=1}^{N_{f}}N_{r}^{q}, \end{array}$$

where $N_{t}^{П}$ and $N_{r}^{П}$ denote the number of transmitting and reception nodes in Stage 2, respectively. $N_{t}^{Ш}$ and $N_{r}^{Ш}$ denote the number of transmitting and receiving nodes in Stage 3, respectively. Similarly, $N_{t}^{q}$ and $N_{r}^{q}$ represent the number of transmitting and receiving nodes in the qth forwarding of Stage 3, respectively. $N_{f}$ denotes the number of forwarding iterations. In Stage 2, $N_{s}$ nodes are selected to broadcast, thus $N_{t}^{П}=N_{s}$. Because the receiving nodes in Stage 3 forward their packet with the probability $p_{2}$, $N_{r}^{q-1}$ and $N_{t}^{q}$ satisfy the following:

(22) $$N_{t}^{q}=N_{r}^{q-1}\cdot p_{2}.\;$$

When $N_{t}^{q*}=N_{r}^{q*-1}\cdot p_{2}\leq 0$, no node forwards packets and the forwarding process is completed. Thus, $N_{f}=q^{*}-1$, $N_{t}^{Ш}=\sum_{q=1}^{N_{f}}N_{t}^{q},N_{r}^{Ш}=\sum_{q=1}^{N_{f}}N_{r}^{q}$. Additionally, $N_{r}^{0}=N_{r}^{П}$ and $N_{t}^{0}=N_{s}$. Next, we formulate the expression of $N_{r}^{П}$, $N_{t}^{q}$ and $N_{r}^{q}$.

### 4.1. Formulating $N_{r}^{П}$

**Proposition** **1.** *The number of receptions in Stage 2 $N_{r}^{П}$ is:*

(23) $$N_{r}^{П}=N_{s}N\pi r_{t}^{2}-C_{N_{s}}^{2}\pi^{2}Nr_{t}^{4}.\;$$

**Proof.**  According to the procedures of Stage 2, $N_{r}^{П}$ equals the number of neighboring nodes around all the source nodes $N_{s,nei}$ minus the number of nodes $N_{r2}$ located in the overlapping communication region of the two sources nodes. This relation occurs because each node receives just one packet and the number of receptions for those nodes is counted twice, thus $N_{r}^{П}$ can be represented as follows:

(24) $$N_{r}^{П}=N_{s,nei}-N_{r2}.\;$$

The average number of neighboring nodes for all source nodes $N_{s,nei}$ is expressed as follows:

(25) $$N_{s,nei}=N_{s}N\frac{\pi r_{t}^{2}}{S}=N_{s}N\pi r_{t}^{2}.\;$$

**Figure 5:**
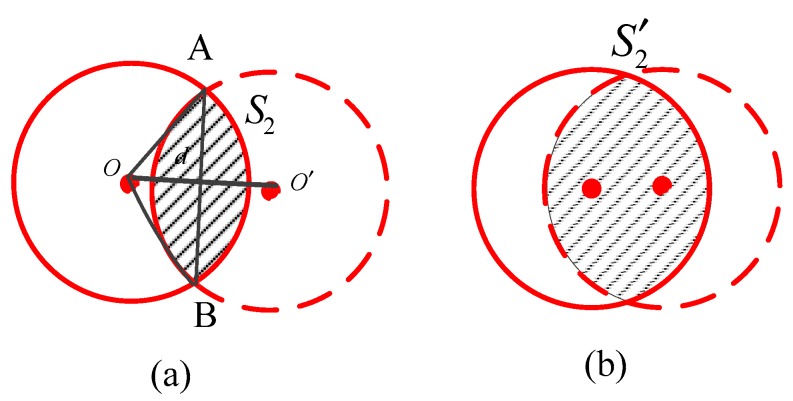
Diagram of two communication nodes: (**a**) $r_{t}<d\left(O,O^{\prime }\right)\leq 2r_{t}$; and (**b**) $0<d\left(O,O^{\prime }\right)\leq r_{t}$.

In Figure 5, the red circle denotes the communication region and $S_{2}$ represents the shaded area jointly covered by the two source nodes. A and B are two intersections. When the distance between two source nodes $d\left(O,O^{\prime }\right)$ satisfies $0<d\left(O,O^{\prime }\right)\leq 2r_{t}$, $N_{r2}$ exists. Thus, the probability $p_{L}$ of an existing communication between the two nodes is expressed as follows:

(26) $$p_{L}=p\left\{d\left(O,O^{\prime }\right)\leq 2r_{t}\right\}=\frac{\pi {\left(2r_{t}\right)}^{2}}{S}=4\pi r_{t}^{2}.\;$$

In the $N_{s}$ source nodes, an average of $N_{L}$ nodes pairs satisfy the condition in Equation (26) (i.e., $N_{L}$ source nodes pairs can communicate with each other). The expressions for $N_{L}$ and $N_{r2}$ are, respectively, as follows:

(27) $$N_{L}=C_{N_{s}}^{2}\cdot p_{L}=C_{N_{s}}^{2}\cdot 4\pi r_{t}^{2}.\;$$

(28) $$N_{r2}=N_{L}\times N\times \frac{{\bar{S}}_{2}}{S}.\;$$

Because the nodes are uniformly distributed and $0<d\left(O,O^{\prime }\right)\leq 2r_{t}$, the probability p{d≤x} is equal to

(29) $$F_{1}\left(x\right)=p\left\{d\leq x\right\}=\frac{\pi x^{2}}{\pi {\left(2r_{t}\right)}^{2}}=\frac{x^{2}}{4r_{t}^{2}}.\;$$

Thus, the probability density function (PDF) $f_{1}\left(x\right)$ is

(30) $$f_{1}\left(x\right)=F_{1}{}^{\prime }\left(x\right)=\frac{x}{2r_{t}}.\;$$

In this case, the area $S_{2}/2$ equals the area of sector OAB minus the area of triangle OAB:

(31) $$\begin{array}{l} S_{2}=2\left(\frac{r_{t}^{2}}{2}\times 2\arccos\frac{d}{2r_{t}}-\frac{1}{2}\times \frac{d}{2}\times 2\sqrt{r_{t}^{2}-\frac{d^{2}}{4}}\right)\\ \;=2r_{t}^{2}\arccos\frac{d}{2r_{t}}-\frac{d}{2}\sqrt{4r_{t}^{2}-d^{2}}. \end{array}$$

Thus, the expected area of $S_{2}$ is calculated as follows:

(32) $${\bar{S}}_{2}=\int_{0}^{2r_{t}}S_{2}f_{1}\left(x\right)dx=\int_{0}^{2r_{t}}\left(2r_{t}^{2}\arccos\frac{x}{2r_{t}}-\frac{x}{2}\sqrt{4r_{t}^{2}-x^{2}}\right)\frac{x}{2r_{t}^{2}}dx\;=\frac{\pi }{4}r_{t}^{2}.\;$$

Combining Equations (27), (28) and (32), $N_{r2}$ can be formulated as:

(33) $$N_{r2}=N_{L}\times N\times \frac{{\bar{S}}_{2}}{S}=C_{N_{s}}^{2}\cdot 4\pi r_{t}^{2}\cdot N\cdot \frac{\pi }{4}r_{t}^{2}=C_{N_{s}}^{2}\pi^{2}Nr_{t}^{4}.\;$$

Finally, we substitute Equations (25) and (33) into Equation (24), to obtain the representation of $N_{r}^{П}$:

(34) $$N_{r}^{П}=N_{s}N\pi r_{t}^{2}-C_{N_{s}}^{2}\pi^{2}Nr_{t}^{4}.\;$$

### 4.2. Formulating $N_{r}^{q}$

**Figure 6:**
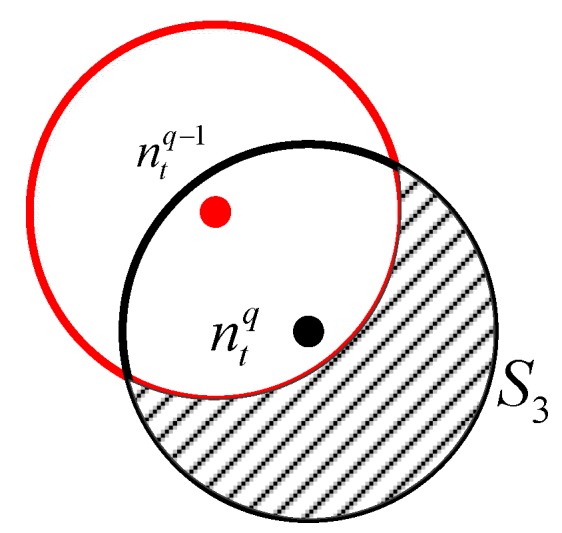
Diagram of forwarding packets.

Figure 6 shows the forwarding procedure of Stage 3, where $n_{t}^{q}$ denotes the transmitting node in the qth forwarding; its communication range is represented by the black circle. Node $n_{t}^{q-1}$ broadcasts its packet in the (q−1)th forwarding process. Because of the reception conditions, the nodes located in area $S_{3}$ can receive the forwarded packet broadcast by $n_{t}^{q}$. Let $N_{r1}^{q}$ denotes the number of receiving nodes in area $S_{3}$.

**Figure 7:**
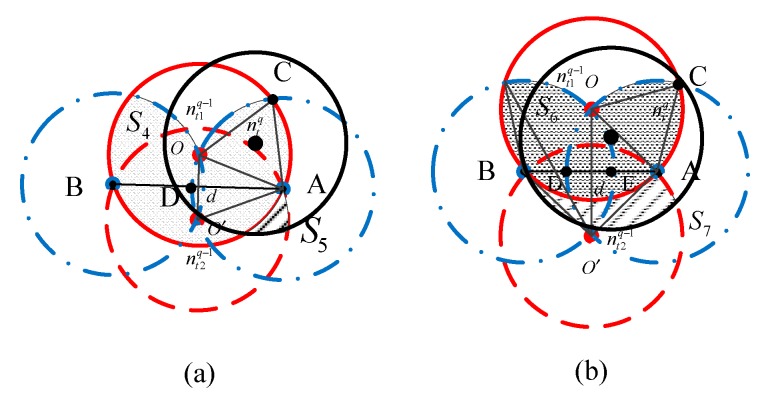
The diagram of Case 1: (**a**) $0<d\left(n_{t1}^{q-1},n_{t2}^{q-1}\right)<r_{t}$; and (**b**) $r_{t}<d\left(n_{t1}^{q-1},n_{t2}^{q-1}\right)<2r_{t}$.

**Figure 8:**
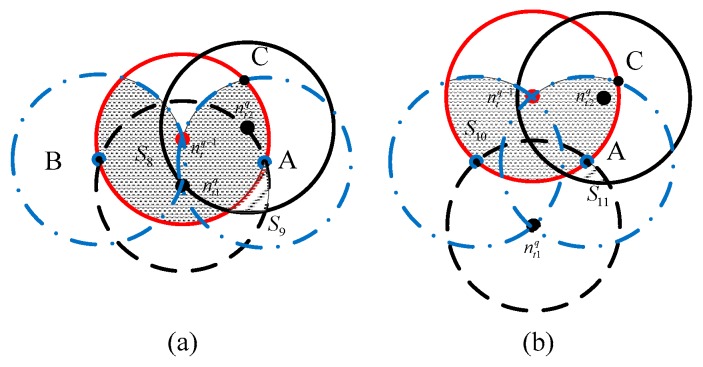
The diagram of Case 2: (**a**) $0<d\left(n_{t1}^{q-1},n_{t2}^{q-1}\right)<r_{t}$; and (**b**) $r_{t}<d\left(n_{t1}^{q-1},n_{t2}^{q-1}\right)<2r_{t}$.

Besides, there are two situations should be considered:

1. Case 1: As presented in Figure 7, there are two broadcasting nodes $n_{t1}^{q-1}$ and $n_{t2}^{q-1}$ in the (q−1)th forwarding, while their communication ranges are represented by the two red circles. This case can be divided into two situations via the distance $d\left(n_{t1}^{q-1},n_{t2}^{q-1}\right)$: (a) $0<d\left(n_{t1}^{q-1},n_{t2}^{q-1}\right)<r_{t}$; and (b) $r_{t}<d\left(n_{t1}^{q-1},n_{t2}^{q-1}\right)<2r_{t}$. Taking the first situation as an example, if node $n_{t}^{q}$ is located in the black area $S_{4}$, the nodes in the shadow area $S_{5}$ can receive packets from nodes $n_{t}^{q}$ or $n_{t2}^{q-1}$, thus the number of receptions of those nodes is counted twice, and that value should be subtracted from $N_{r1}^{q}$. Suppose that the number of receiving nodes in areas such as $S_{5}$ is $N_{r2}^{q}$ and that the number in areas such as $S_{7}$ is $N_{r3}^{q}$.
2. Case 2: Similarly, there are two transmitting nodes $n_{t1}^{q}$ and $n_{t2}^{q}$ in the qth forwarding, whose communication ranges are represented by two black circles in Figure 8. This case can be divided into two situations via the distance $d\left(n_{t1}^{q},n_{t}^{q-1}\right)$: (a) $0<d\left(n_{t1}^{q},n_{t}^{q-1}\right)<r_{t}$; and (b) $r_{t}<d\left(n_{t1}^{q},n_{t}^{q-1}\right)<2r_{t}$. Taking the first situation as an example, when node $n_{t2}^{q}$ is distributed in the black area $S_{8}$, the nodes located in shadow area $S_{9}$ receive one of the packets broadcasted by node $n_{r1}^{q}$ or $n_{r2}^{q}$. Thus, the number of receptions for those nodes is counted twice, which should be subtracted from $N_{r1}^{q}$. Suppose that the number of reception nodes in areas such as $S_{9}$ is $N_{r4}^{q}$ and the number in areas such as $S_{10}$ is $N_{r5}^{q}$.

In conclusion, the number of receptions $N_{r}^{q}$ for Stage 3 in the $q^{th}$ forwarding can be expressed as follows:

(35) $$N_{r}^{q}=N_{r1}^{q}-N_{r2}^{q}-N_{r3}^{q}-N_{r4}^{q}-N_{r5}^{q}.\;$$

Next, we formulate the expression of $N_{r1}^{q},N_{r2}^{q},N_{r3}^{q},N_{r4}^{q}$ and $N_{r5}^{q}$.

#### 4.2.1. Calculating $N_{r1}^{q}$

As shown in Figure 6, the nodes in shadow area $S_{3}$ would receive the packet. Thus, $N_{r1}^{q}$ is calculated as follows:

(36) $$N_{r1}^{q}=N_{t}^{q}\times N\times \frac{{\bar{S}}_{3}}{S}.\;$$

In the above formula, ${\bar{S}}_{3}$ can be expressed as follows:

(37) $${\bar{S}}_{3}=\pi r_{t}^{2}-{\bar{S^{\prime }}}_{2}.\;$$

Because the nodes are uniformly distributed and $0<d\left(O,O^{\prime }\right)\leq r_{t}$, the probability p{d≤x} equals

(38) $$F_{2}\left(x\right)=p\left\{d\leq x\right\}=\frac{\pi x^{2}}{\pi r_{t}^{2}}=\frac{x^{2}}{r_{t}^{2}}.\;$$

Thus, the PDF $f_{2}\left(x\right)$ is

(39) $$f_{2}\left(x\right)={F^{\prime }}_{2}\left(x\right)=\frac{2x}{r_{t}^{2}}.\;$$

Combining Equations (31), (37) and (39), we obtain

(40) $${{\bar{S}}^{\prime }}_{2}=\int_{0}^{r_{t}}\left(2r_{t}^{2}\arccos\frac{x}{2r_{t}}-\frac{x}{2}\sqrt{4r_{t}^{2}-x^{2}}\right)\frac{2x}{r_{t}^{2}}dx=\left(\pi -\frac{\sqrt{3}}{4}\right)r_{t}^{2}.\;$$

(41) $${\bar{S}}_{3}=\pi r_{t}^{2}-{\bar{S^{\prime }}}_{2}=\pi r_{t}^{2}-\left(\pi -\frac{3\sqrt{3}}{4}\right)r_{t}^{2}=\frac{3\sqrt{3}}{4}r_{t}^{2}.\;$$

Thus, we obtain

(42) $$N_{r1}^{q}=N_{t}^{q}N\frac{3\sqrt{3}}{4}r_{t}^{2}.\;$$

#### 4.2.2. Calculating $N_{r2}^{q}$

Next, we formulate the expression of $N_{r2}^{q}$. As presented in Figure 7a, the value of $N_{r2}^{q}$ is the number of receive node in area $S_{5}$, thus we have

(43) $$N_{r2}^{q}=C_{N_{t}^{q-1}}^{2}\times {p^{\prime }}_{L}\times N_{t}^{q}\times \frac{{\bar{S}}_{4}}{\pi r_{t}^{2}}\times N\times \frac{{\bar{S}}_{5}}{S},\;$$

where ${\bar{S}}_{4}$ denotes the expected area of the black region, ${\bar{S}}_{5}$ denotes the expected area of the shadow region, and ${p^{\prime }}_{L}$ denotes the probability that the distance between two nodes satisfies $0\leq d\left(O,O^{\prime }\right)\leq r_{t}$. Thus, we have the following:

(44) $${p^{\prime }}_{L}=p\left\{d\left(O,O^{\prime }\right)\leq r_{t}\right\}=\frac{\pi r_{t}{}^{2}}{S}=\pi r_{t}^{2}.\;$$

As shown in Figure 7a, the area $S_{4}$ equals twice the area of region ACD minus the half intersection area of circle A and B, i.e., ${\bar{S}}_{2}/2$, plus the half intersection area of circle O and $O^{\prime }$, i.e., ${{\bar{S}}^{\prime }}_{2}/2$. The area of region ACD equals to the area of sector ACD plus the area of sector OAC minus the area of triangle OCA; thus,

(45) $$S_{ACD}=\pi r_{t}^{2}\times \frac{\frac{\pi }{3}+\arcsin\left(\frac{d}{2r_{t}}\right)}{2\pi }+\pi r_{t}^{2}\times \frac{\frac{\pi }{3}}{2\pi }-\frac{1}{2}\times r_{t}\times \frac{\sqrt{3}r_{t}}{2}=\frac{r_{t}^{2}}{2}\left[\frac{\pi }{3}+\arcsin\left(\frac{d}{2r_{t}}\right)\right]+\frac{\pi }{6}r_{t}^{2}-\frac{\sqrt{3}}{4}r_{t}^{2}.\;$$

(46) $$S_{4}=S_{ACD}-\frac{{\bar{S}}_{2}}{2}+\frac{{\bar{S^{\prime }}}_{2}}{2}\;=\frac{r_{t}^{2}}{2}\left[\frac{\pi }{3}+\arcsin\left(\frac{d}{2r_{t}}\right)\right]+\frac{\pi }{6}r_{t}^{2}-\frac{\sqrt{3}}{4}r_{t}^{2}-\frac{{\bar{S}}_{2}}{2}+\frac{{\bar{S^{\prime }}}_{2}}{2}.\;$$

Combining Equations (39) and (46), we obtain

(47) $$\begin{array}{l} {\bar{S}}_{4}=\int_{0}^{r_{t}}2\times \left[\frac{r_{t}^{2}}{2}\left(\frac{\pi }{3}+\arcsin\frac{x}{2r_{t}}\right)+\frac{\pi r_{t}^{2}}{6}-\frac{\sqrt{3}r_{t}^{2}}{4}\right]f_{2}\left(x\right)dx-\frac{\pi r_{t}^{2}}{8}+\frac{1}{2}\left(\pi -\frac{3\sqrt{3}}{4}\right)r_{t}^{2}\\ \;=\frac{\pi r_{t}^{2}}{2}-\frac{\pi r_{t}^{2}}{8}+\frac{1}{2}\left(\pi -\frac{3\sqrt{3}}{4}\right)r_{t}^{2}=\frac{7\pi r_{t}^{2}}{8}-\frac{3\sqrt{3}r_{t}^{2}}{8}, \end{array}$$

where $2\int_{0}^{r_{t}}x\arcsin\frac{x}{2r_{t}}dx=\left(\frac{\sqrt{3}}{2}-\frac{\pi }{6}\right)r_{t}^{2}$. According to the method in [28], we can get the approximate value of ${\bar{S}}_{5}$, i.e., ${\bar{S}}_{5}≃S_{5,\max}=\pi r_{t}^{2}/6$. Finally, combining Equations (43), (44) and (47), we obtain

(48) $$N_{r2}^{q}=\frac{C_{N_{t}^{q-1}}^{2}NN_{t}^{q}}{6}\left(\frac{7\pi }{8}-\frac{3\sqrt{3}}{8}\right).\;$$

#### 4.2.3. Calculating $N_{r3}^{q}$

The expression of $N_{r3}^{q}$ is similar to that of $N_{r2}^{q}$:

(49) $$N_{r3}^{q}=C_{N_{t}^{q-1}}^{2}\times p_{L}\times N_{t}^{q}\times \frac{{\bar{S}}_{6}}{\pi r_{t}^{2}}\times N\times \frac{{\bar{S}}_{7}}{S},\;$$

As shown in Figure 7b, because $r_{t}\leq d\left(O,O^{\prime }\right)\leq 2r_{t}$, ${\bar{S}}_{6}$ is calculated as follows:

(50) $$\begin{array}{l} {\bar{S}}_{6}=2S_{ACD}=\int_{r_{t}}^{2r_{t}}2\times \left[\frac{r_{t}^{2}}{2}\left(\frac{\pi }{3}+\arcsin\frac{x}{2r_{t}}\right)+\frac{\pi r_{t}^{2}}{6}-\frac{\sqrt{3}r_{t}^{2}}{4}\right]f_{1}\left(x\right)dx\\ \;=\int_{r_{t}}^{2r_{t}}\frac{\pi }{3}xdx+\frac{1}{2}\int_{r}^{2r}x\arcsin\frac{x}{2r_{t}}dx-\frac{\sqrt{3}}{4}\int_{r}^{2r}xdx\\ \;=\left(\frac{13\pi }{24}-\frac{\sqrt{3}}{2}\right)r_{t}^{2}, \end{array}$$

where $\frac{1}{2}\int_{r}^{2r}x\arcsin\frac{x}{2r_{t}}dx=\frac{\pi }{24}r_{t}^{2}-\frac{\sqrt{3}}{8}r_{t}^{2}$, and $S_{7}$ is expressed as

(51) $${\bar{S}}_{7}={\bar{S}}_{2}-{\bar{S}}_{12},\;$$

where ${\bar{S}}_{12}$ is the intersection area of circle O and circle $O^{\prime }$ when $r_{t}\leq d\left(O,O^{\prime }\right)\leq 2r_{t}$, thus

(52) $${\bar{S}}_{12}=\int_{r_{t}}^{2r_{t}}\left(2r_{t}^{2}\arccos\frac{x}{2r_{t}}-\frac{x\sqrt{4r_{t}^{2}-x^{2}}}{2}\right)\frac{x}{2r_{t}^{2}}dx\;=\frac{3\sqrt{3}r_{t}^{2}}{16},\;$$

and

(53) $${\bar{S}}_{7}={\bar{S}}_{2}-{\bar{S}}_{12}=\left(\frac{\pi }{4}-\frac{3\sqrt{3}}{16}\right)r_{t}^{2}.\;$$

Combining Equations (49), (50) and (53), we obtain

(54) $$N_{r3}^{q}=\frac{C_{N_{t}^{q-1}}^{2}4r_{t}^{2}NN_{t}^{q}}{\pi }\left(\frac{13}{24}-\frac{\sqrt{3}}{2\pi }\right)\left(\frac{\pi }{4}-\frac{3\sqrt{3}}{16}\right).\;$$

#### 4.2.4. Calculating $N_{r4}^{q}$ and $N_{r5}^{q}$

As illustrated in Figure 8, the two black circles denote the communication range of two transmitting nodes, $n_{t1}^{q}$ and $n_{t2}^{q}$, in the q forwarding. The red circle denotes the communication range of transmission node $n_{t}^{q-1}$ in the q−1 forwarding. The calculation of $N_{r4}^{q}$ and $N_{r5}^{q}$ is similar to that of $N_{r2}^{q}$ and $N_{r3}^{q}$:

(55) $$N_{r4}^{q}=C_{N_{t}^{q}}^{1}C_{N_{t}^{q-1}}^{1}{p^{\prime }}_{L}\times C_{N_{t}^{q}-1}^{1}\frac{{\bar{S}}_{8}}{\pi r_{t}^{2}}\times N\frac{{\bar{S}}_{9}}{S},\;$$

(56) $$N_{r5}^{q}=C_{N_{t}^{q}}^{1}C_{N_{t}^{q-1}}^{1}p_{L}\times C_{N_{t}^{q}-1}^{1}\frac{{\bar{S}}_{10}}{\pi r_{t}^{2}}\times N\frac{{\bar{S}}_{11}}{S},\;$$

where $S_{8}$ and $S_{10}$ denote the area of the black region and $S_{9}$ and $S_{11}$ denote the area of the shadow region. Compared with Figure 7 and Figure 8, we have the following:

(57) $$\left\{ \begin{array}{l} {\bar{S}}_{8}={\bar{S}}_{4},{\bar{S}}_{9}={\bar{S}}_{5}\\ {\bar{S}}_{10}={\bar{S}}_{6},{\bar{S}}_{11}={\bar{S}}_{7} \end{array}\right..\;$$

Thus, the expressions of $N_{r4}^{q}$ and $N_{r5}^{q}$ are:

(58) $$N_{r4}^{q}=\frac{N_{t}^{q}N_{t}^{q-1}\pi Nr_{t}^{4}\left(N_{t}^{q}-1\right)}{6}\left(\frac{7\pi }{8}-\frac{3\sqrt{3}}{8}\right)\;$$

(59) $$N_{r5}^{q}=4N_{t}^{q}N_{t}^{q-1}N\pi r_{t}^{4}\left(N_{t}^{q}-1\right)\left(\frac{13}{24}-\frac{\sqrt{3}}{2\pi }\right)\left(\frac{\pi }{4}-\frac{3\sqrt{3}}{16}\right)\;$$

In conclusion, by combining Equations (35), (42), (48), (54), (58) and (59), we can obtain the expression of $N_{r}^{q}$:

(60) $$\begin{array}{l} N_{r}^{q}=N_{t}^{q}N\frac{3\sqrt{3}}{4}r_{t}^{2}-\frac{C_{N_{t}^{q-1}}^{2}NN_{t}^{q}}{6}\left(\frac{7\pi }{8}-\frac{3\sqrt{3}}{8}\right)-\frac{C_{N_{t}^{q-1}}^{2}4r_{t}^{2}NN_{t}^{q}}{\pi }\left(\frac{13}{24}-\frac{\sqrt{3}}{2\pi }\right)\left(\frac{\pi }{4}-\frac{3\sqrt{3}}{16}\right)\\ \;-\frac{N_{t}^{q}N_{t}^{q-1}\pi Nr_{t}^{4}\left(N_{t}^{q}-1\right)}{6}\left(\frac{7\pi }{8}-\frac{3\sqrt{3}}{8}\right)-4N_{t}^{q}N_{t}^{q-1}N\pi r_{t}^{4}\left(N_{t}^{q}-1\right)\left(\frac{13}{24}-\frac{\sqrt{3}}{2\pi }\right)\left(\frac{\pi }{4}-\frac{3\sqrt{3}}{16}\right). \end{array}$$

### 4.3. The Formulation of $N_{Ttot}$ and $N_{Rtot}$

**Theorem** **1.** *Assume that all N sensor nodes are deployed randomly and uniformly in a distributed WSNs with a boundary length of 1, and each node has a transmission range of*$r_{t}$*. If we gather data based on CS-DDSG scheme, then*$N_{Ttot}$*and*$N_{Rtot}$*are, respectively, expressed as follows:*

(61) $$N_{Ttot}=N_{s}+\sum_{q=1}^{N_{f}}N_{t}^{q},\;$$

(62) $$\begin{array}{cc} N_{Rtot} & =N_{s}N\pi r_{t}^{2}-C_{N_{s}}^{2}\pi^{2}Nr_{t}^{4}+\sum_{q=1}^{N_{f}}N_{t}^{q}N\frac{3\sqrt{3}}{4}r_{t}^{2}-\sum_{q=1}^{N_{f}}\frac{C_{N_{t}^{q-1}}^{2}NN_{t}^{q}}{6}\left(\frac{7\pi }{8}-\frac{3\sqrt{3}}{8}\right)\\ & -\sum_{q=1}^{N_{f}}\frac{C_{N_{t}^{q-1}}^{2}4r_{t}^{2}NN_{t}^{q}}{\pi }\left(\frac{13}{24}-\frac{\sqrt{3}}{2\pi }\right)\left(\frac{\pi }{4}-\frac{3\sqrt{3}}{16}\right)-\sum_{q=1}^{N_{f}}\frac{N_{t}^{q}N_{t}^{q-1}\pi Nr_{t}^{4}\left(N_{t}^{q}-1\right)}{6}\left(\frac{7\pi }{8}-\frac{3\sqrt{3}}{8}\right)\\ & -\sum_{q=1}^{N_{f}}4N_{t}^{q}N_{t}^{q-1}N\pi r_{t}^{4}\left(N_{t}^{q}-1\right)\left(\frac{13}{24}-\frac{\sqrt{3}}{2\pi }\right)\left(\frac{\pi }{4}-\frac{3\sqrt{3}}{16}\right), \end{array}$$

*where*$N_{r}^{0}=N_{r}^{П},N_{t}^{0}=N_{s}$*,*$N_{t}^{q}=N_{r}^{q-1}\times p_{1}$*and*$N_{f}=q^{*}-1$*, where*$q^{*}$satisfies$N_{t}^{q^{*}}=N_{r}^{q^{*}-1}\times p_{1}\leq 0$*. The expression for*$N_{r}^{П}$*is given in Equation (34)*.

**Proof.**  As presented in the above derivation, we can obviously obtain Equation (61) based on Equation (21) and the correlative description in Stage 2 of CS-DDSG. Furthermore, by combining Equations (21), (34) and (60), we can obtain the expression of Equation (62).  □

## 5. Performance Evaluation and Analysis

To evaluate the effectiveness of CS-DDSG, we ran simulations in MATLAB 2012b. The simulation parameters were set as shown in Table 1. Furthermore, we adopted the FFT orthonormal basis and the orthogonal matching pursuit (OMP) method for the reconstruction algorithm. We used the real sensor readings extracted from the GreenOrbs [29] system.

In this paper, we present the performance comparations of CS-DDSG, Compressive Sensing Data storage (CStorage) [20], Improved CStorage (ICStorage) [21], Compressed Network Coding based Distributed data Storage (CNCDS) [21] and Direct Cluster-Based Compressive Sensing Data Collection (DCCS) [15] on unreliable links. These first four schemes all combine DDS and CS to gather data. CStorage, ICStorage and CNCDS are concerned with reducing the number of transmission and fusions. In CStorage, intermediate nodes receive the broadcasting packets when they first receive, and then, they forward the received packet with a given probability. The intermediate nodes in ICStorage forward their own readings rather than the received source nodes readings. In the CNCDS scheme, the intermediate nodes receive broadcast packets only if the receiving node does not share any node IDs with the corresponding transmitting node. We also analyze the numbers of transmissions, receptions and fusions involved in the first four algorithms. DCCS combines CS and cluster topology to reduce the total power consumption with no consideration of packet loss rate. All member nodes gather data and transmit to cluster heads, where the CS measurements and measurement matrices are generated and send to sink directly. Additionally, we discuss the impact of packet loss rate, the number of measurements and the proportion of source nodes on the performance of CS-DDSG. The simulation results shown are the average values from 1000 runs.

**Figure 9:**
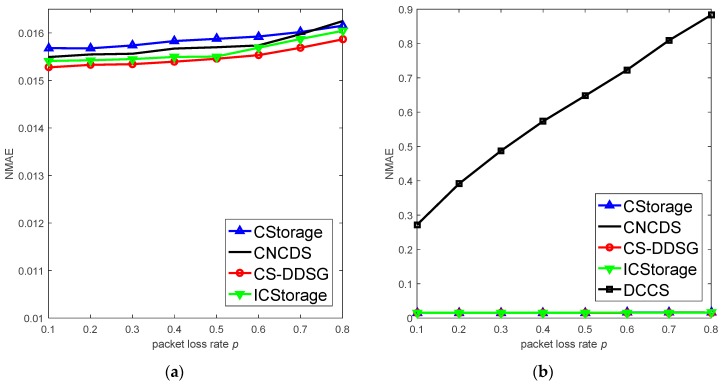
Performance of the different algorithms in unreliable links: (**a**) DDS-based algorithms; and (**b**) comparison between DDS-based algorithms and cluster-based algorithm.

First, we evaluate the performance on unreliable links when p1=0.3,M=50, as shown in Figure 9. It can be seen that: (1) As p increases, the reconstruction accuracy of all the algorithms decreases in Figure 9a. When p≤0.6, the NMAEs of the four algorithms are stable and increase gradually, which indicates that CS-DDSG is effective at resisting the packet loss. Although the packet loss rate impacts the nodes receiving broadcasting packets, the sink still gathers enough packets to recover the data. In addition, the sink constructs the measurement matrix based on received packets, which avoids the need to measure the lost nodes and reduces the impact of unreliable links on measurement vector Y. However, the performance of DCCS is poor with an increase in p. Sink cannot find the lossy nodes and still reconstructs data based on the original measurement matrices. Thus, DCCS is sensitive to p. (2) CS-DDSG outperforms the other algorithms. This improved performance occurs because in CS-DDSG, nodes receive only one packet which is broadcasted by its neighbor nodes in CS-DDSG. Thus, the measurement vectors have the characteristic of strong spatial correlation, which is utilized by CS to recover the data. However, in the other algorithms, nodes would fuse packets from distant nodes as long as the receipt condition is satisfied, which leads to a weak spatial correlation of measurement vectors. Thus, CS-DDSG outperforms the other algorithms.

**Figure 10:**
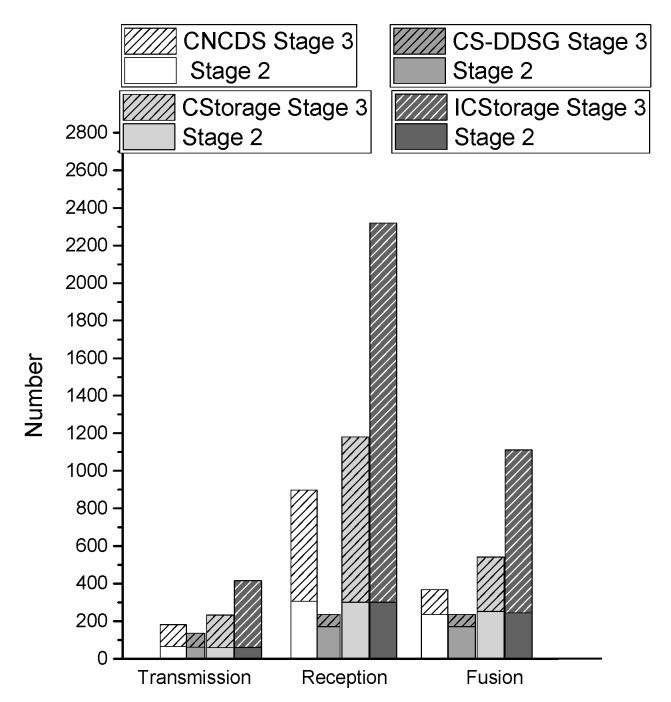
The total number of transmissions, receptions and fusions in Stages 2 and 3.

We present the total number of transmissions, receptions and fusions of the four algorithms in Figure 10 when p=0.3 and $p_{1}=0.15$. CS-DDSG requires fewer transmissions, receptions and fusions than do the CNCDS, CStorage and ICStorage schemes. This is because nodes in CS-DDSG receive packets only the first time and broadcast their packets with the probability $p_{2}$, after which then they do not receive any data. However, for CNCDS, CStorage and ICStorage, nodes continue to receive packets as long as the reception condition is satisfied. CStorage and ICStorage in particular focus on reducing the number of transmissions. Moreover, compared with CNCDS, CStorage and ICStorage, CS-DDSG scheme reduces $N_{Ttot}$ by up to 23.9%, 42.5% and 67.8%, respectively, and reduces $N_{Rtot}$ by up to 73.8%, 80.2% and 89.9%, respectively.

**Figure 11:**
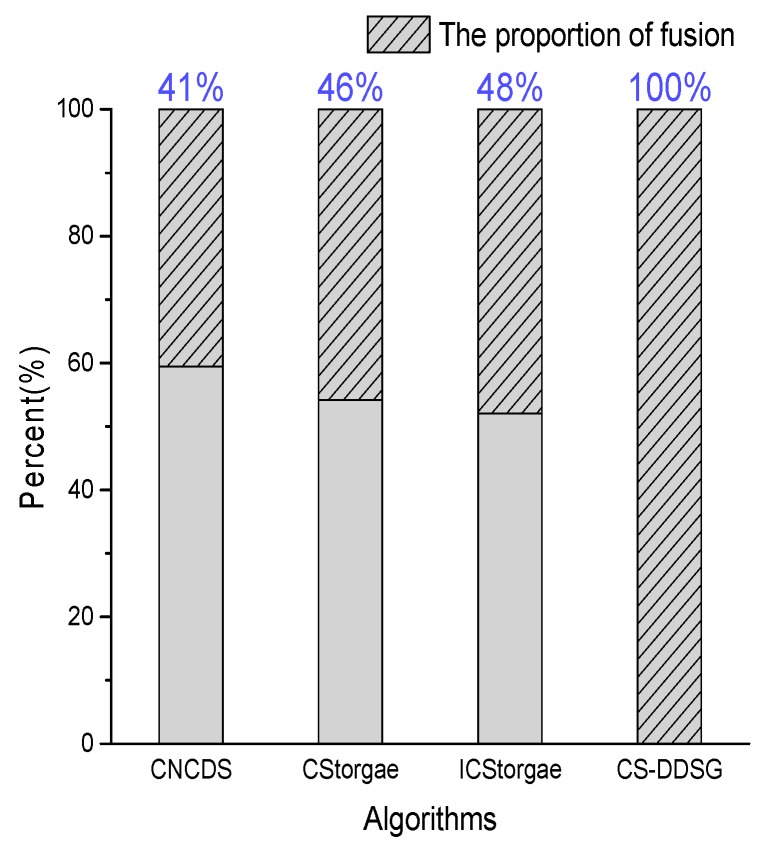
The fusion proportion of the total number of receptions.

Furthermore, we investigate the fusion proportion of the total number of receptions. As presented in Figure 11, only 41% of the receiving nodes in CNCDS merge the received packets; the authors consider that only 41% of nodes lose energy. In fact, 59% of the receiving nodes also consume energy because they would receive the broadcast packet first and then determine whether the condition of CNCDS are satisfied; the received packets will be merged only if they satisfy the condition. Thus, energy is consumed even when the received packets are not fused. However, the number of receptions in [21] is the same as the number of fusions, which is less counted. Similarly, 46% and 48% of the receiving reception nodes in CStorage and ICStorage merge the packets, respectively. In CS-DDSG, all received nodes are fused and no redundancy occurs because the nodes receive packets only once. Thus, the energy consumption of CS-DDSG receiving nodes is much smaller than that of the other algorithms. In conclusion, CS-DDSG effectively reduces both the number of transmissions and receptions.

**Figure 12:**
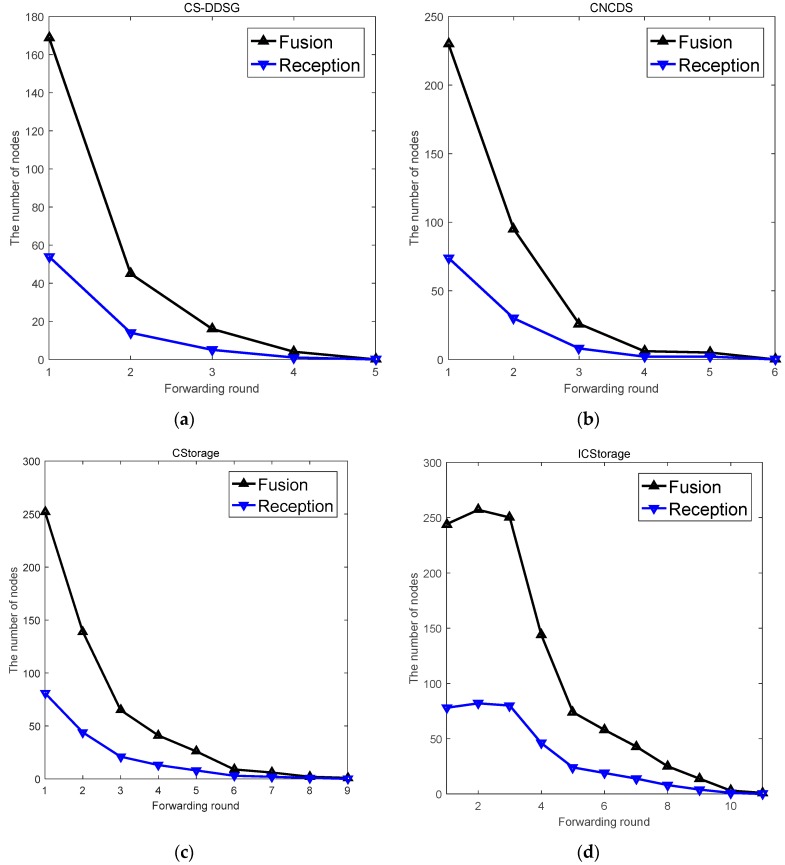
The number of fusions and receiving nodes during the forwarding process: (**a**) CS-DDSG; (**b**) CNCDS; (**c**) CStorage; and (**d**) ICStorage.

Figure 12 presents the number of fusions and receiving nodes during each forwarding round when $p_{1}=0.15$. The forwarding process of CS-DDSG repeats five times, until no node remains to accept the broadcast packets, while CNCDS, CStorage and ICStorage repeat six, nine and twelve times, respectively. The network employing CS-DDSG has the fastest convergence and characteristics of efficiency due to the strictest reception conditions. Moreover, most of the data fusion occurs during Stage 2, and subsequently the number of fusions rapidly decreases in Stage 3 except in ICStorage.

**Figure 13:**
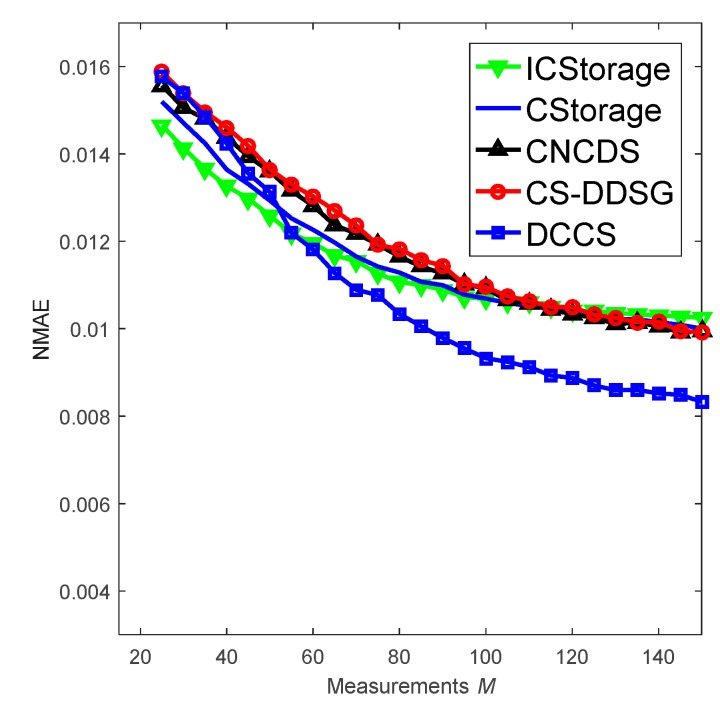
Performance of the algorithms when $p_{1}=0.3$.

In Figure 13, we investigate the recovery performance of the algorithms when $p_{1}=0.3$ and the number of measurements M, which is queried by the mobile sink, ranges from 15 to 150. It can be observed that, with an increase in M, the recovery accuracy of ICStorage, CStorage, CNCDS and CS-DDSG are improved and equivalent, while the performance of CS-DDSG becomes slightly better when M≥100. This improvement occurs because the more information that is gathered, the better is the reconstruction accuracy. According to Equation (12), the sink constructs measurement matrix Φ based on the packets fused by the forwarding nodes. The forwarding nodes of CS-DDSG receive only one packet, and the Φ is sparser than that in the others algorithms. Consequently, less information is gathered and fewer nodes contribute to data recovery for CS-DDSG. However, with an increase in M, more information is gathered and the gaps separating the four algorithms decrease. When M>100, CS-DDSG outperforms the four DDS-based algorithms due to the strong spatial correlation of the measurement vector. Moreover, the reconstruction accuracy of DCCS is the best when M is large enough and there is no packet loss. All nodes in DCCS participate in gathering data and DCCS adopts dense measurement matrix in clusters. Thus, more information is gathered. In addition, performance tends to be stable as M increases.

**Figure 14:**
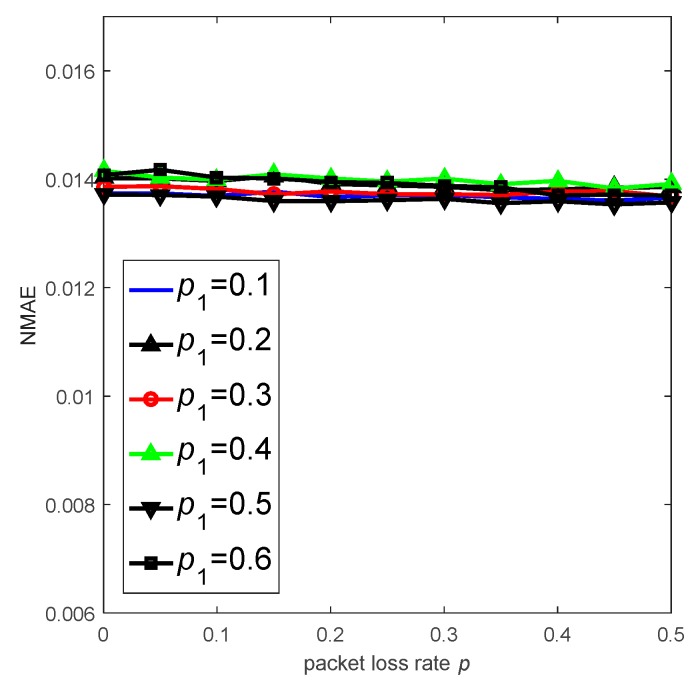
Performance of CS-DDSG with different $p_{1}$ and p.

Figure 14 shows the performance of CS-DDSG under different packet loss ratios p and probabilities $p_{1}$ when M=40. As p increases, the value of NMAE remains stable, i.e., NMAE≈0.014. This result indicates that CS-DDSG effectively resists the packet loss and maintains high reconstruction accuracy even when unreliable links exist. Additionally, its accuracy is not influenced by $p_{1}$ due to the very sparse measurement matrix.

**Figure 15:**
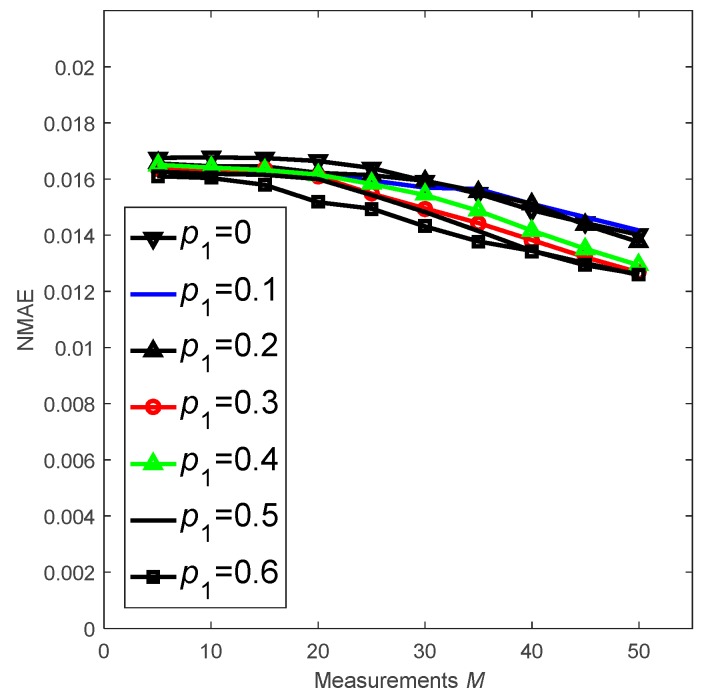
Performance of CS-DDSG with different value of $p_{1}$ and M.

Finally, we investigate how the proportion of source nodes $p_{1}$ impact the recovery accuracy in Figure 15. The simulation results show the following: (1) When $p_{1}=0.4$, the value of NMAE decreases as M increases because more nodes participate in data reconstruction as M increases. (2) When M is fixed, CS-DDSG performance is improved and the trend of NMAE values is very close to the value of $p_{1}$, varying from 0 to 0.6. This effect occurs because, when there are more source nodes, more nodes will receive broadcast packets before the sink obtains data. Hence, the amount of information used for reconstruction increases. However, due to the reception condition, the measurement matrix Φ is sparse. Thus, information is increasingly limited. As a result, the trends of the NMAE values are close to the different value of $p_{1}$.

## 6. Conclusions

In this paper, the data gathering problem is investigated in lossy WSNs using the simple but efficient proposed CS-DDSG algorithm that combines CS theory and DDS. Compared with other correlative and mainstream strategies, CS-DDSG balances the energy consumption and reconstruction performance effectively. In our proposed algorithm, nodes are selected to be source nodes with the probability $p_{1}$ to broadcast their packets. The neighboring nodes around the source nodes receive the broadcasting nodes and update their own packets, which are broadcasted with the probability $p_{2}$. Then, all receiving nodes forward their updated packets with the probability $p_{2}$. The process will be repeated a few times until there are no receiving nodes. Each receiving node receives only one packet. In this way, the numbers of transmissions and fusions are reduced, and the CS reconstruction accuracy is guaranteed. Moreover, the expression of the total number of transmissions and receptions is formulated via RGG. The simulation results and analysis validate that CS-DDSG outperforms the other algorithms in unreliable links.

In addition, we investigate how the measurements M, the packet loss p and the probability $p_{1}$ influence the performance of CS-DDSG. In future research, we plan to explore the possibility of temporal correlations of node readings. Another potential extension of this work is to more strictly demonstrate that the measurement matrix satisfies the RIP.

## Figures and Tables

**Table 1:** Default Simulation Parameters.

	Parameters	Value
N	The total number of sensors	400
a	Boundary length	1
$p_{2}$	The probability of forwarding in Stage 3	0.32
$r_{t}$	Communication radius	0.075

## Nomenclature

WSNs	Wireless Sensor Networks
CS	Compressive Sensing
IoT	Internet of Things
RIP	Restricted Isometry Property
FFT	Fast Fourier Transform
OMP	Orthogonal Matching Pursuit
DDS	Distribute Data Storage
NMAE	Normalized Mean Absolute Error
RGG	Random Geometric Graph
PDF	Probability Density Function
